# A Dynamic Systems Framework for Gender/Sex Development: From Sensory Input in Infancy to Subjective Certainty in Toddlerhood

**DOI:** 10.3389/fnhum.2021.613789

**Published:** 2021-04-09

**Authors:** Anne Fausto-Sterling

**Affiliations:** Department of Molecular Biology, Cell Biology, and Biochemistry, Brown University, Providence, RI, United States

**Keywords:** gender/sex, infancy, dynamic systems, sensory input, subjective outcome, interdisciplinary consortium

## Abstract

From birth to 15 months infants and caregivers form a fundamentally intersubjective, dyadic unit within which the infant’s ability to recognize gender/sex in the world develops. Between about 18 and 36 months the infant accumulates an increasingly clear and subjective sense of self as female or male. We know little about how the precursors to gender/sex identity form during the intersubjective period, nor how they transform into an independent sense of self by 3 years of age. In this Theory and Hypothesis article I offer a general framework for thinking about this problem. I propose that through repetition and patterning, the dyadic interactions in which infants and caregivers engage imbue the infant with an embodied, i.e., sensori-motor understanding of gender/sex. During this developmental period (which I label Phase 1) gender/sex is primarily an intersubjective project. From 15 to 18 months (which I label Phase 2) there are few reports of newly appearing gender/sex behavioral differences, and I hypothesize that this absence reflects a period of developmental instability during which there is a transition from gender/sex as primarily inter-subjective to gender/sex as primarily subjective. Beginning at 18 months (i.e., the start of Phase 3), a toddler’s subjective sense of self as having a gender/sex emerges, and it solidifies by 3 years of age. I propose a dynamic systems perspective to track how infants first assimilate gender/sex information during the intersubjective period (birth to 15 months); then explore what changes might occur during a hypothesized phase transition (15 to 18 months), and finally, review the emergence and initial stabilization of individual subjectivity-the period from 18 to 36 months. The critical questions explored focus on how to model and translate data from very different experimental disciplines, especially neuroscience, physiology, developmental psychology and cognitive development. I close by proposing the formation of a research consortium on gender/sex development during the first 3 years after birth.

## Introduction

### Overview

By 3 years of age, most children-at least those who grow up in Western Educated Industrialized, Rich, Democratic i.e., WEIRD cultures (Henrich et al., 2010)- express a subjective gender/sex identity (see section “A Note on the Meaning and Use of Gender/Sex”). Researchers infer this identity and measure its strength by examining preferences and behaviors understood in WEIRD societies to be more or less typical of boys compared to girls (Zucker, 2005; Zucker and Wood, 2011). Caregivers control infant gender/sex expression by choosing clothing, hairstyles and jewelry on their child’s behalf, but by age three, children enact significant agency in choice of toys, clothing, and playmates (Todd et al., 2018). Gender/sex-related toy preferences do not appear until sometime during the 2nd year. For example, in one multi-age cross-sectional study, researchers found that infants showed no visual preference when shown matched pairs of vehicles and dolls, but that by 18 months toddlers showed a gender/sex-biased preference for these items and the girls in the study associated certain toys with a particular gender/sex (Serbin et al., 2001). Gender/sex self-knowledge and concomitant preferences and behaviors appear in bits and pieces over time. In another study of 2 year olds, 67 percent could label themselves as their assigned gender/sex although they were less successful at similarly labeling other children (54 percent), toys (23 percent), or activities (13 percent) (Campbell et al., 2002). This sequence suggests that self-identity at least partially precedes the understanding and/or enactment of gender/sex-differentiated preferences and behaviors (Ruble et al., 2010). As one additional example, using a longitudinal design of 17 and 21 month olds, researchers noted that at both ages, toddlers had gender/sex related preferences for play with trucks and dolls. They had no gender/sex related preferences, however, for other stereotyped activities such as tea sets, brush and comb sets and blocks. The differences, present at 17 months, increased in size by 21 months. In this study the investigators related the acquisition of gender/sex category words such as “boy” and “girl” to the increase in differences in play preferences between 17 and 21 months (Zosuls et al., 2009).

How can we explain this acquisition of gender/sex subjectivity which seems to be absent before about 15 months, but that apparently snaps into place during the next 9 months, and stabilizes during the third year of development? Two theoretical approaches to understanding the strength of gender/sex identity at age three predominate in the research literature. The first emphasizes biological underpinnings. Based on studies of children who had been exposed to unusually high levels of androgens or estrogens during fetal development, or studies that correlate levels of amniotic hormones and later play behavior, or studies of identity formation in children with a severe intersex condition called cloacal exstrophy, a number of researchers have concluded that fetal hormonal environments contribute strongly to gender/sex identity development (Collaer and Hines, 1995; Hines et al., 2002; Reiner and Kropp, 2004; Reiner, 2005; Auyeung et al., 2009; Lillard, 2015). More recently, even the authors of some of these earlier papers linking hormones and gender/sex development have acknowledged the weakness of the evidence supporting a strong theory (direct and/or linear) of hormonal causation of gender/sex differences in childhood behavior (Jordan-Young, 2010; Hines et al., 2016; Xiong et al., 2020). Strong conclusions from other authors still exist, though. For example one recent publication claimed that “Gender identity is biologically conferred during the middle trimester of pregnancy” and referred to “gender identity … as biological, innate, and immutable” p. 33 (O’Hanlan et al., 2018).

Beyond the sketchiness of the evidence, however, I find a “biological underpinnings” approach deeply unsatisfying mainly because it does not tell a developmental story. Studies infer or directly measure hormone levels at time A, and then often years later assess some aspect of behavior (time B), as if the events of time A and the behaviors of time B are directly and linearly linked, while the events of the intervening years remain unmentioned and apparently irrelevant to the outcome.

The second predominating theoretical approach comes from researchers in developmental, cognitive, and social learning psychology. These scholars offer a more nuanced narrative. Ruble, Martin, and Berenbaum (Ruble et al., 2006) discussed the possible causes of developmental change, as seen through the combined lenses of biology, cognitive development, and socialization theory. Building on the earlier work of Huston (1983, 1985), Ruble et al. presented a matrix of constructs. The matrix included biological, behavioral and cultural versus content areas cross-referenced with “biological gender,” “activities and interests,” “personal-social attributes,” “gender-based social relationships,” and “stylistic and symbolic content.” The assembly and organization of this large body of work was heroic and laid a necessary foundation for the ideas I present in this article, but their approach still uses a static theoretical framework. First, most publications within this body of research seem to consider gender/sex identity to be a fixed “thing” apparently located somewhere in the body or brain [see section “Discussion” in Fausto-Sterling et al. (2020)]. And, even though this literature offers a developmental timeline and more nuanced details of when components of this “thing” appear, in my opinion, this body of work does not have a working theory that interweaves the constructs presented in little compartments in the “matrix of gender-typing” table (p. 859) into a narrative of dynamic and continuous development.

Some of the authors cited in the previous paragraphs now recognize dynamic systems theory as an important theoretical and research approach. Hines, for example, noted that “One appealing aspect of a developmental systems perspective is that it can obviate the misleading nature versus nurture debate.” p. 35 (Hines, 2015) while Martin and Ruble emphasize that dynamic systems theory provides “more nuanced views of gender at different timescales.” By timescales they intend on the one hand explaining long term developmental changes (time scale of years) in gender identity from infancy to childhood, adolescence and adulthood, and on the other hand describing how gender plays out in short term interactions (time scale of minutes). But to date these authors have not taken on the challenge of outlining a multi-level, dynamic, and developmental systems theory of early gender/sex development.

A wealth of publications in the biosciences and psychology use and advocate for dynamic systems theory (DST) (Oyama, 1985, 2000; Smith and Thelen, 1993a, b). Several key ideas–self organization, complexity, embodiment, continuity in time and dynamic stability–lie at the heart of DST (Thelen, 2005). *Self organization*, a well-known phenomenon in biology, refers to the apparently spontaneous emergence of pattern or order due to the stabilization of internal processes, rather than an external directive force. Self-organizing systems are often complex, heterogeneous, and encompass multiple levels of biological and social organization (Kelso, 1995; Warren, 2006). Their study requires understanding short-term (e.g., neural events of a specific memory formation, or the visual and vocal interactions between a caregiver and child during a brief exchange), mid-term (the development of coordinated play–see for example de Barbaro et al., 2013b) – and long-term dynamics (see Thelen, 2000 for an integrated discussion of short, mid and long term dynamics). In one discussion of *complexity*, Thelen wrote “Human behavior is the product of many interacting parts that work together to produce a coherent pattern under particular task, social and environmental constraints” (p. 261) (Thelen, 2005). Behaviors, in this conceptualization are not caused by a single driving force–be it hormones or parental directive–but are the collective property of a complex system.

Dynamic systems theorists often discuss *embodiment* as a critical component of complexity. An embodied phenomenon is one that “emerges in the interaction of an organism with an environment … as a result of sensory-motor activity.” (p. 278) (Smith, 2005). Thelen wrote that embodied cognition emerges from and remains enmeshed within the body’s interaction with the world (Thelen, 2000). By extension, I theorize that gender/sex identity “depends on the kinds of experiences that come from having a body with particular perceptual and motor capabilities that are inseparably linked and that together form the matrix within which reasoning, memory, emotion, language, and all other aspects of mental life are embedded” (p. 5) (Thelen, 2000). In this formulation, gender/sex is not an abstract thing that resides somewhere in the mind or brain, but is a dynamic process that emerges from in-the-moment experiences over time. Once in a state of *dynamic stability* gender/sex identity seems independent and unattached from the processes that produced it. But prior to achieving such stability any emerging system may experience instability as it transits from a previously stable state to a new and different one. Change here is *non-linear* and involves a measurable *phase shift* (Thelen and Ulrich, 1991; Thelen and Smith, 2006). A central feature of DST is that new behaviors are linked *continuously in time* to older phenomena and that to understand the origins of a behavior of interest, one must start before it exists, watch it emerge and figure out the key systems components involved in its production.

### A Note on the Meaning and Use of Gender/Sex

The terms sex and gender imply an additive causal model that is (biology plus culture) usually with some allowance for “interaction” as a third, poorly articulated term. Unger and Crawford pointed out the difficulty with the sex versus gender terminology when they wrote “With the possible exception of very specific reproductive behaviors, however, it is not possible to determine how much of a particular trait or behavior is influenced by biological versus social factors…” (p. 124) (Unger and Crawford, 1993).

Responding to such conceptual difficulties, in a research project that focused on hormones, which are most often listed as a feature of “sex,” van Anders and Dunn introduced the term gender/sex “because,” they wrote, “differences cannot knowingly be attributed to biology or gender socialization” (p. 207) (van Anders and Dunn, 2009). van Anders defined gender/sex as pertaining to “whole people/identities and/or aspects of women, men and people that relate to identity and/or cannot really be sourced specifically to sex or gender” [Table 2 in van Anders (2015)]. Fausto-Sterling, Kaiser, and Pitts-Taylor used the term sex/gender to connote body-based characteristics that are shaped by gendered social interactions (Kaiser et al., 2007; Fausto-Sterling, 2012; Kaiser, 2012; Pitts-Taylor, 2016). However, to maintain consistency with the way the term was initially introduced, I now prefer the term *gender/sex*. Furthermore, in mainstream psychology the term has begun to catch on (Hyde et al., 2018).

### The Phases of Gender/Sex Development

Gender/sex development bears the hallmarks of a dynamic system. In previous work, based on a review of developmental psychology literature, my colleagues and I divided gender/sex emergence into three phases (Fausto-Sterling, 2020; Fausto-Sterling et al., 2020). The first, which spans the period from birth (or before) through about 14 months, involves the acquisition of gender/sex recognition skills. For example, between the ages of 6 to 8 months infants demonstrate the ability to distinguish between male and female voice recordings. By 9 months they can differentiate pictures of male from those of female faces. These skills, a compilation of which may be found in Fausto-Sterling et al. (2012), reflect an increasing ability to recognize and remember repeated elements in the environment. The second is a period of instability during which the infant assimilates earlier embodied learning during a period when he or she also acquires language and independent mobility. As described in the opening paragraph, gender/sex preferences start to appear but are difficult to measure. During the third phase, identity, measurable by specific behaviors and preferences, and the ability to indicate group belonging becomes evident and stable. In the next three sections of this essay I lay out some of the known parameters for each of these phases.

#### Phase 1

**FIGURE 1:**
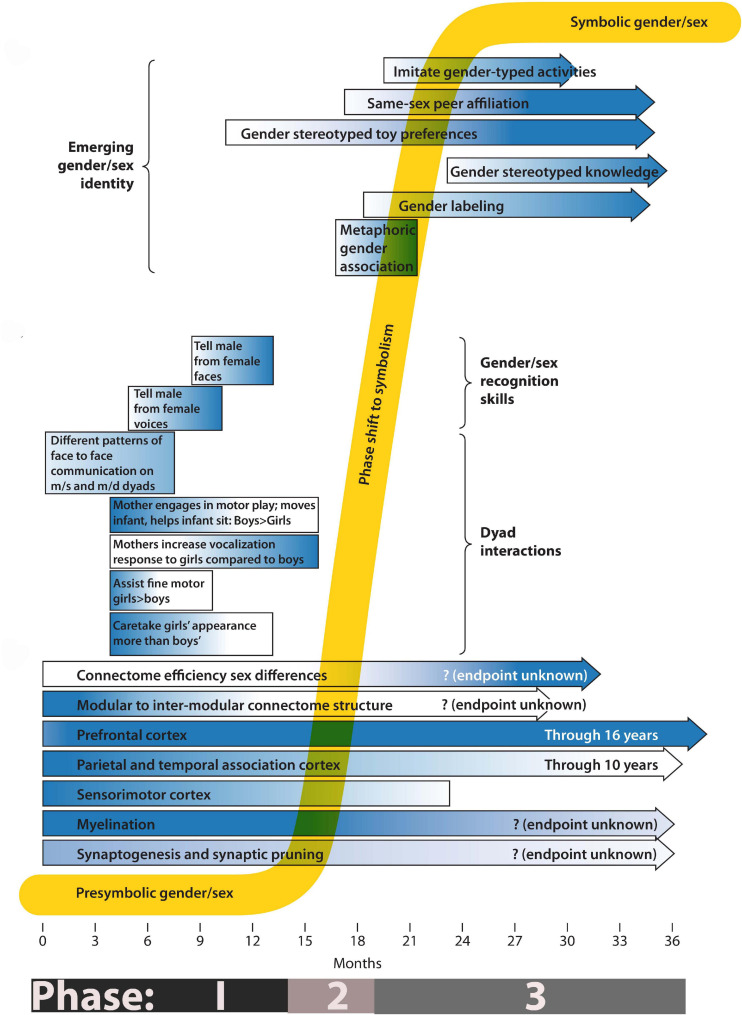
Phases of gender/sex development. The composite timeline illustrates systems undergoing change from birth to 3 years. Longitudinal bars show gradients of activity, from light (low activity) to dark (high activity). The lower third of the drawing emphasizes known changes in the growth and development of the nervous system. The middle third indicates the timing of gender/sex skill acquisition and of known gender/sex differences in care-giver/infant dyad interactions. The top third indicates the emergence of behaviors associated with subjective gender/sex identity. For a definition and measure of connectome efficiency please refer to reference (DiPietro and Voegtline, 2015).

I have argued (Fausto-Sterling 2019) that sensorimotor experiences register in the body, both as cognitive and neuromuscular memory (Fausto-Sterling, 2019) and make this argument more explicitly in Figure 1 of the current essay. During Phase 1 the infant’s motor and sensory development integrates the data set available for recognizing and embodying gender/sex. For example, at 3–5 months, when infants lie on their backs, or are held, facing in or facing out, by an adult, or are bathed and diapered, they experience touch from another person more frequently than they voluntarily reach out and touch an object, surface or person. On the caregiver side of the dyad, features of touch such as frequency, duration, pressure, and association with speech or play, etc. may differ depending on the gender/sex of the person who touches and on the perceived gender/sex of the infant. On the infant side, babies can differentiate between adult male and female voices and faces.

Thus, a baby’s sensory input depends on more than its own sensorimotor abilities. Indeed, during the period of physical helplessness, adults provide most of the visual, touch, and sound input. Even at a very young infant age, this provision is differentiated by gender/sex. We have reported (Fausto-Sterling et al., 2020), for example, that mothers of 3-month old infants engage in motor social play with sons for longer durations than daughters, that they help sons sit more frequently than daughters, and that they shift sons’ positions more frequently and for longer time periods than daughters.” We also found that mothers of 3–6 month old sons touched them more frequently than did mothers of daughters (Fausto-Sterling et al., 2015), and reported a sex by age interaction (for months 3–12) for both instrumental touch (e.g., moving a child from one spot to another) and stimulatory touch. Finally, we and others have reported perceived sex of infant-differentiated frequencies of infant-directed maternal vocalization (Sung et al., 2013; Johnson et al., 2014).

In sum, gender/sex embodiment in the first 14 months involves the dynamics of self-development of motor and sensory skills, their use in absorbing the experiential data presented to the infant, and the creation of the environment by primary caregivers. This creation involves the physical setting (room décor, toys, and clothing) but also the intimacies of physical touch, imposed movement, and sound. Later in this article I will return to Phase 1 to recount what researchers in the disciplines of neuroscience, physiology and developmental psychology already know from their particular disciplinary point of view about development more generally and gender/sex embodiment more specifically.

#### Phase 2

**FIGURE 2:**
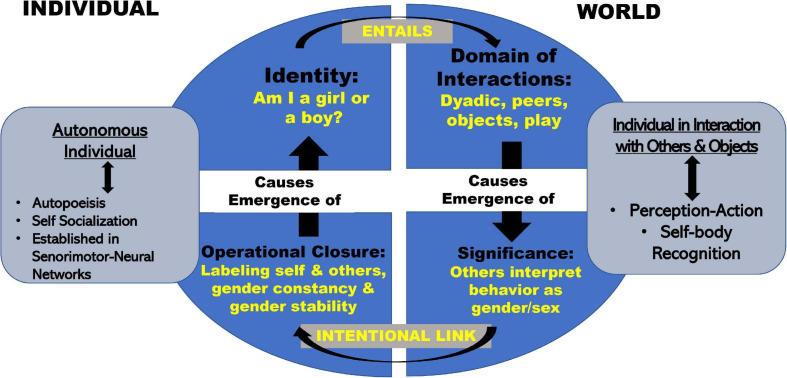
Model of embodied development of gender/sex.

Between approximately 15 and 18 months I hypothesize that the infant shifts (Phase 2) from recording intersubjectively generated, presymbolic gender/sex knowledge, to producing embodied, symbolically understood and expressed gender/sex identity. There are fewer reported findings about gender/sex development in the time slice between 15 and 18 months see especially (Fausto-Sterling et al., 2012; Figure 2) a result, probably, of increased variability during this time frame. In this S-shaped trajectory, Phase 1 represents a period in which the underpinnings of subjective gender/sex-related sensory and cognitive data slowly accrete; but gender/sex itself is not visible by any measures at researchers’ disposal. I hypothesize that Phase 2, entails a relatively chaotic period when high individual variability and a disruption of the stable period of presymbolic accretion makes measuring group differences quite difficult (Thelen, 2005). Starting at about 18 months, however, a subjective sense of gender/sex and the preferences and behaviors that accompany that sense start to emerge. Phase 3, which continues to at least 3 years (and actually beyond, but not covered in this paper), is the period during which subjective gender/sex stabilizes and deepens.

#### Phase 3

Between 18 and 36 months toddlers consolidate and stabilize gender/sex self-knowledge and gender/sex knowledge of the world. Their sense of self as having a gender/sex identity becomes internalized, although intersubjective feedback and stabilization contributes to identity throughout the life cycle. From 18 months on children express gender/sex knowledge symbolically, for example, via a pink/blue color scheme and clothing or play preferences designated within a culture as gender/sex differentiated (Eichstedt et al., 2002). Ruble, Lurye, and Zozuls write about the rigidity with which some children in the 3–6 years old age range insist on using gender symbols and preferences such as clothing, hair style, friendships and play styles. Ruble and colleagues associate acquiring gender/sex specific language with this active period of gender/sex self socialization. “Girls’ love of pink, frilly dresses” they write “may be viewed as a kind of obsession linked to developing knowledge about social categories.” (p. 4) (Ruble et al., 2010). JeongMee Yoon’s Pink and Blue Project provides one artist’s vision of this obsession (Yoon, 2005).

### Organization of This Paper

In the section entitled “The Challenges: Synthesizing Theories and Investigatory Approaches to Explicating Gender/Sex Identity Development”, I review some of the things we do–and do not–know about underlying systems which most likely support gender/sex emergence, and discuss the challenges of understanding how infants integrate events that occur on different time scales and at different levels of biological organization. I also review some of what we know about how the infant itself integrates sensory and social inputs *en route* to becoming an independent subject. Finally, I suggest possible approaches to surmounting the challenges researchers face in synthesizing myriad theories and empirical approaches to the study of early gender/sex development.

## Studying Gender/Sex as a Dynamic System Requires Interdisciplinarity

### Disciplines and Biological Scale

Figure 1 summarizes some of the processes important to understanding gender/sex formation during the first 3 years of development. At birth, or even prenatally (Moon et al., 2013), infants record sensorimotor information in a “non-verbal, imagistic, acoustic, visceral, or temporal mode” (Beebe and Lachmann, 1994) (p. 132). The information diagrammed in Figure 1 describes events and processes that occur at markedly different levels of biological scale. During the first few months of Phase 1, for example, notable changes take place at the cellular and intercellular level (bars indicating specific aspects of brain development). Complex parental behaviors and developing infant cognition–which occur at higher levels of organization than the establishment of inter-neuronal and neuro-muscular connectivity-become features of the middle and later parts of Phase 1. During Phase 2 I propose that infants integrate the different levels of information acquired during Phase 1, allowing qualitatively new traits to appear. During Phase 3 a full presentation of self as having a specific gender/sex appears. This self-presentation draws on cultural symbols such as hair and clothing styles, toy preferences, etc. It seems sudden and new. However, as a developmental systems theorist, I view it as an emergent property, a qualitative shift that results from the quantitative accumulation (at the cellular, intercellular, inter-organ, and intersubjective levels) of body knowledge about gender/sex. Figure 1 serves as a guide for the discussion of relevant physiological, dyadic and autonomous behaviors that mark the presymbolic, transitional, and symbolic phases of gender/sex development. I note that I have drawn on a literature that comes almost exclusively from studies of white, middle class, European-origin families, and that patterns of gender/sex-related behaviors and interests discussed here are not universal (Lew-Levy et al., 2020).

#### Neuroscience

Neuroscientist Lise Eliot wrote, “Toy play may look instinctive in children–as when we see toddlers cuddling a doll or pushing a toy truck across the floor–but every piece of such actions requires learning and tuning of neural circuits to the specific sensory, motor, spatial, social, cultural, and motivational demands of both object and environment” (p. 171) (Eliot, 2018). To explore Eliot’s remark, I selectively review findings from the neurosciences that are relevant to the presymbolic embodiment of gender/sex.

Even before birth, synaptic connections involved with sensory and related motor activities proliferate exuberantly. As neurons attain peak bushiness, they gain specificity by pruning some connections and strengthening others (Elman et al., 1996). Nervous transmission also becomes more efficient and accurate when long nerve fibers gain electrical insulation via myelination. As represented in the bottom third of Figure 1, synaptogenesis and synaptic pruning is especially active during the first year of development. The first 6 months are particularly important for the development of the sensorimotor, prefrontal, parietal, and association cortices (Thompson and Nelson, 2001), while intense myelination occurs during the first year of infancy. [For an overview of human brain development see Zelazo et al. (2010)]. Critical to the idea that gender/sex–at least initially–is a process requiring both dyadic interactions and interactions between infants and objects in its world is the fact that specific synaptic connections form under the influence of specific experiences. While synaptic plasticity and experience-related myelination (Forbes and Gallo, 2017) are well demonstrated in animal models, it is more difficult to perform exacting experiments in humans (Marshall, 2015; Mansvelder et al., 2019). Nevertheless, using non-invasive measurements of brain activity has lead researchers to state unequivocally that “The experiences children have literally shape their brains” [p. 3 (Meltzoff and Kuhl, 2016)]. Parsons et al., review the substantial literature on postnatal brain development in human infants including the critical importance to brain development of social and sensory stimuli (Parsons et al., 2010).

Despite the well accepted overview of events in the developing infant brain, it is difficult to directly link anatomy with specific behaviors and functions (Gao et al., 2009, 2017). Parsons et al. (2010) have published a timeline that correlates infant age with emerging abilities (e.g., face-processing at 2–8 months or joint attention at 14–18 months) and they note what brain regions seem to be associated with these abilities (Parsons et al., 2010). Knowledge of such associations increasingly derive from the identification of neural circuits/networks. Identifying circuits and higher order networks, and assigning to them specific roles in brain function and emergent behavior is an area of active research and theoretical consideration (Friston, 2011; Kelso et al., 2013). Findings to date suggest that primary sensorimotor and visual areas are more fully developed, but that systems such as the limbic, frontoparietal (attentional, problem-solving, working memory), and the default network are highly variable among individuals at birth (Xu et al., 2018), decline in activity but develop more fully by the end of the first year. Xu and colleagues (2018) suggest that this pattern of initial high variability may be due to what they call a lower memory load before birth.

We know little about gender/sex structural differences in the central nervous systems of infants and children. Although Giedd et al., documented differences in brain structure between boys and girls as young as 4 years old (Giedd et al., 1997; Gogtay et al., 2004; Lenroot et al., 2007), no data exist for infants and toddlers. Perhaps, though, there is a story to be explored at the level of nervous system functioning and the connectome. One publication reported no differences between males and females in the efficiency of either global or local information transfer in two-week olds and 1-year olds. However, both local and global brain network efficiency was reported to be significantly greater in male compared to female 2-year olds (Yap et al., 2011), the time point that marks the end of my proposed phase transition to symbolism, and the early expression of differences in preferences and behaviors. This finding is thought-provoking, but comes from a single study on a small sample, and thus requires replication and expansion. Even then it will remain to be seen if any gender/sex differences in connectome function relate to the behaviors and preferences that at age three reveal subjective identity formation.

#### Physiology

For reasons of space and clarity, Figure 1 does not cover physiological development. But gaining control of autonomic physiological functions such as temperature regulation, sleep cycles, and states of arousal is a significant task facing young infants. The development of physiological self-regulation has been extensively studied (Feldman, 2003, 2007; DiPietro and Voegtline, 2015). Porges and Furman (2011) describe a time line for the early development of the autonomic (vagal) nervous system which initially uses feeding (visceral) circuits to regulate basic functions such as respiration, but by 6 months regulates autonomic states by social engagement (Porges and Furman, 2011).

Of particular interest are findings of infant and parent gender/sex related differences of arousal-stimulating parental behaviors. Parental play stimulation differences according to infant sex in early infancy seem only rarely to have been studied (Zosuls and Ruble, 2018). Instead, with the exception of Korner’s analysis (Korner, 1974), the existing literature focuses on bonding and dyad synchrony, defined as a correlation between one partner’s behavior and the other’s response within a defined period of time (usually on the order of milliseconds or seconds). For example, Feldman (2003) studied levels of synchrony and patterns of arousal (using a 3-level arousal scale in which high arousal was positive and energetic) in mother-daughter, mother-son, father-daughter and father-son dyads in 5-month-old infants. She observed that same-sex dyads achieved greater synchrony than other-sex dyads. Fathers’ play sessions tended to reach a peak of arousal one or more times per session. This contrasted with mothers, who in 44% of the play sessions with daughters and 35% for sons had no arousal peak.

#### Developmental Psychology/Cognitive Development

##### Gender/sex recognition skills

As depicted in Figure 1, Phase 1 includes the appearance of cognitive skills that we have named gender/sex recognition skills. For example, at 5 and 7 months infants cannot categorically distinguish between male and female faces, but by 9 months they have acquired this skill (Leinbach and Fagot, 1993). They have, by then, also gained the ability to associate female voices with female faces (Poulin-Dubois et al., 1994). One study found that preference for male or female faces in five-month olds varied with the sex of the primary caregiver (Quinn et al., 2002). While researchers collect connectomic data using magnetic resonance brain scans, they use visual or aural habituation studies to collect information about prelinguistic, cognitive gender/sex skills. I am presuming that these operate at a scale above the level of the connectome, but that they reflect connectome function. Designing new studies that look for the emergence of gender/sex recognition skills while measuring specific brain activity, using methods that combine qualitative microanalysis at multiple levels of dyad interaction with quantitative assessment of “action arcs” over developmental time, could inform us about what areas of the brain become involved with gender recognition (moving from midscale to microscale analysis) (Kuhl and Rivera-Gaxiola, 2008; Ra̧czaszek-Leonardi et al., 2019).

### Phase 1: Dyad Interactions

Many behaviors relevant to Phase 1 involve interactions within an infant-caregiver dyad. These interactions may focus directly on one another or they may involve the infant and caregiver jointly interacting with an object such as a toy or bottle. During the first three months, for example, infants and caregivers spend a great deal of time in face-to-face communication. In one study, after the initial month, mothers, and infants communicated face to face for longer periods when the infant was on the sofa. But during month three, girls spent longer periods than boys in face to face communication when being held in their mothers’ arms (see Phase 1 level B in Figure 1 of this essay) (Lavelli and Fogel, 2002). During the first 6 months there are other findings of gender/sex differences in dyadic interactions. Fausto-Sterling et al. (2015) reported that from months 3 to 6, compared to mothers with sons, mothers of daughters more frequently adjusted their child’s appearance by combing her hair, and straightening her dress or repositioning a hair ribbon or barrette. During this same time period mothers vocalized more to daughters than to sons (Sung et al., 2013). In a last example, another analysis showed that from months 3 to 6, mothers engaged in more gross motor activities with sons than with daughters and shifted sons from one position to another with greater frequency and duration than they did daughters (Fausto-Sterling et al., 2020).

What, if any, might be the effects of such differences in sensory input (during Phase 1) on the transition (during Phase 2) that results in the consolidation of subjective gender/sex (in Phase 3)? It seems likely that gender/sex differentiation of self and others is indirect, that is, it results from repeated observations and sensory interactions rather than direct instruction. If a caregiver regularly hands a plushie baseball and glove to a six-month old boy, for example, and he later expresses a desire to throw a ball, that desire does not emerge because he has received the instruction that boys throw balls. Rather it emerged within a meshwork of dyadic and triadic interactions. At 4 months, for example, infants mostly look at or manipulate a single object, usually held by the caregiver. Between 6 and 9 months, infants divide their attention between objects they themselves are holding and objects held onto by their caregiver (de Barbaro et al., 2015). By 12 months triadic attention between an adult play partner, the baby and one or more play objects has become fairly elaborate, and involves complex social exchanges (de Barbaro et al., 2013a). Throughout, caregivers offer attention-getting clues, including manipulating an object, gaze shift, and/or verbalization (Deák et al., 2017). It is within the broad sequence of developmental events that the more specific self-definitions of gender/sex emerge. Rather–as has happened up until now- than avoid studying this period because of behavioral instability, a dynamic systems analysis points to Phase 2 as exactly the period that we need to imaginatively investigate.

Such developmental processes will vary individually depending upon the pattern of adult approaches to directing attention, and individual variability in infant sensory systems. For example, my research group produced unpublished data [using the methods described in Fausto-Sterling et al. (2020)] that between 3 and 12 months of infant age, mothers manipulated objects more often and for longer duration if they were part of a mother-daughter dyad compared to a mother-son dyad. What might such a difference in adult behavior mean for the development of infant gender/sex? Does more insistent manipulation promote greater joint attention which in turn becomes a scaffold for more socially interactive patterns of play, a pattern that by age three has emerged as a group difference related to gender/sex? How does the emergence of joint attention skills relate to earlier gender/sex variations in sensory input via speech and person-to-person handling? Such questions, which offer a framework for gender/sex development during Phase 1, await empirical investigation.

One challenge is to understand whether and how the above types of gender/sex differences in dyadic behavior shape the underlying developing nervous system and produce the ability to recognize aspects of gender/sex (what we refer to as gender/sex skills) in the infant’s world. A shift in scale of the relevant events (interactive behaviors between two people or two people and an object at a macro level, inter-constructing with neural networks, connectome structure, and cellular and synaptic connections at a microlevel) is involved. Such macro to micro crossovers require varied disciplinary expertise. In the section entitled “The Challenges: Synthesizing Theories and Investigatory Approaches to Explicating Gender/Sex Identity Development”, I will discuss methods that might enable productive collaborations between scientists with different disciplinary skills and who work on different scales of organismal and inter-organismal organization.

#### How Does an Infant Integrate Levels During Phase 1?

Think of infants as statisticians. Presented with repeated and diverse sensory inputs, they measure the frequencies of sequences of motor, visual, object and linguistic events, extracting “chunks,” i.e., elements that co-occur, which they store in distributed neural networks. As they repeatedly encounter similar chunks, linked elements connect more tightly. It is through these general learning mechanisms and the cellular mechanisms involved with neural plasticity, we hypothesize, that infants extract and stabilize the structures and meanings of gender/sex, first presymbolically, and then via language and symbolism (Mareschal and Quinn, 2001; Balas et al., 2018; Gliga, 2018; Smith et al., 2018).

Smith and colleagues write that “the developing infant creates a curriculum for statistical learning” (p. 1) (Smith et al., 2018). I would modify this assertion to say that the dyad creates the curriculum. Consider videotaped sequences of a dyadic (mother-daughter) interaction collected as described in Fausto-Sterling et al. (2020). When the baby was 2.4 and 3.2 months, the mother washed her child’s head. Throughout each two- to four-minute episode, she encouraged the baby to enjoy the wash, saying “doesn’t that feel good? Do you want to help?” and, as she massaged the soap into her head, “Oh you smell so good.” The baby smiled and tried to participate, which the mother encouraged. In these two chunks the infant combined what appeared to be pleasurable tactile sensations with a maternal narration of events. At 3.4 months, a new element appeared when the mother decided that the baby’s hair was long enough to brush. She brushed gently for 13 s and said “that’s not bad. All done. All done. That’s pretty.” The baby had been sitting on the changing table, and looking around the room, but as the mother said the first “All done,” she looked directly up at her mother’s face as the mother bent over her. In this third chunk, there was again a pleasant tactile sensation on the head, but this time combined with positive dyadic eye contact and maternal patter about how pretty the baby looked. Did the last chunk build on, or interact with and strengthen the preceding ones, while for the first time integrating a gendered comment (“that’s pretty”) into the event network? Was the establishment of a link between pleasurable touch sensations and gender/sex-weighted language underway?

Patterns of infant care structure the data chunks that infants sense and assimilate. And as those patterns become infused with gender/sex, so too does the data set or curriculum made available to the infant. At the level of neural circuits I imagine the following: as predicted by developmental systems theorists, as infants acquire motor skills such as reaching and stepping, they first call on a redundant repertoire of neural circuits (Gottlieb et al., 1998; Nishiyori et al., 2016). As they gain experience through increasingly goal-directed activities, the initially large areas of neural activation become more restricted and refined. Thus, the neural responses that underpin specific motor activities derive from both the specific goal and the experience of pursuing it. Work on multisensory, multimodal processing echoes this general idea that infants process sensory input broadly in early development, but as they gain both sensory and symbolic experience, multi-sensory perception narrows, and becomes more culturally specific (Murray et al., 2016; Cao et al., 2017). Applying these general principles to the acquisition of gender/sex, I hypothesize that at first infants perceive and turn toward any and all caregivers. With time, however, and in response to repeated patterns of care giving (both who and how), gender/sex perception develops and becomes more narrowly specific. This occurs both with regard to expectations of who the caregiver is, but also with regard to how the infant itself is touched and spoken to.

During Phase 1, early versions of cognition are already at work. These too rely on repetition and the context of exposure. The brilliant studies of Rovee-Collier and colleagues demonstrate that infants as young as 3 months can recognize and categorize objects. Furthermore, their object memory and ability to associate categories depend on regular exposure (Galluccio and Rovee-Collier, 1999, 2005; Mareschal and Quinn, 2001; Bhatt et al., 2004). The authors of a recent overview of infant memory note the following: during infancy encoding speed increases and memory duration lengthens, memory retrieval becomes more flexible, and reminders allow the infant to retrieve forgotten memories (Cuevas and Sheya, 2019). These changes “are embedded in broader socio-cultural contexts with shifting ecological demands that are in part determined by the infants themselves” (abstract) (Cuevas and Sheya, 2019). This is the same claim about infant memory that I am making about gender/sex development. Connecting back to the question of toy preference, although it does not stabilize until Phase 3, it seems likely that the kinds and numbers of toys found in an infant’s environment from birth, combined with how (and how often) specific toys are offered by caregivers, and what unprompted interest the infant exhibits, produce presymbolic memory traces that the infant draws on and transforms into cognitive memory and subjective desire during Phases 2 and 3.

Several research groups that study multisensory systems emphasize (Kuhl et al., 2001, 2006; Murray et al., 2016; Lewkowicz et al., 2018) this developmental pattern of proceeding from diffuse to focused processes. This body of work involves connecting faces to specific vocalizations and language recognition. Studies suggest that both before birth and for the first 3 to 6 months after birth, infants exhibit broad, low-level responses to sound and sight stimuli. Over time, responses narrow. At first, an infant may respond fully to a non-native spoken sound; with further sensory experience, the response narrows and becomes native language specific. Lewkowicz and Ghazanfar (2009) suggested that this perceptual narrowing results from the selective elaboration of synapses in specific response to postnatal experience (Lewkowicz and Ghazanfar, 2009). Between the ages of four to five and eight to 12 months, infants develop the abilities to “perceive, learn, and generalize recursive, hierarchical, pattern rules” (p. 1) (Lewkowicz et al., 2018). Does the infant response to gender/sex-related information follow this pattern-broad and inclusive at first, followed by a narrowing introduced by gender/sex specific experiences? If so, what forms and types of gender/sex data are most important for shaping gender/sex identity development?

### From Dyads to Independent Subject: Phases 2 and 3

As infants move from Phase 1 through Phase 2 and into Phase 3, they separate from the dyad and become more independent actors. The increasing precision of motor skills such as crawling, walking and grasping is a critical animator of this separation (Campos et al., 2000). So too is the acquisition of language, which also facilitates the emergence of symbolic thought and actions (Zosuls et al., 2009; Salo et al., 2018). During Phase 2 and early Phase 3, infants transform body memory into cognitive memory. And, as these transitions accumulate during Phase 2, infants (now toddlers) enter (during early Phase 3) into a period of self-stabilization. Finally, once Phase 3 is well underway, the newly independent, subjective and (semi) autonomous sense of self stabilizes via the process of autopoiesis, defined as a network that reproduces itself, “and that also regulates the boundary conditions necessary for its ongoing existence as a network” (p. 327) (Bourgine and Stewart, 2004).

Figure 2 illustrates this dynamically stable state consolidated during Phase 3. The model is based on concepts developed by Varela (1997) and Smith and Gasser (2005). Applying to gender/sex Varela’s idea that individual identities involve interactive domains, a child cannot arrive at a stable sense of self as boy or girl (upper left quadrant: *Identity*) without engaging in dyadic interactions, and specific sorts of gender/sex-specified activities (upper right-hand ellipse quadrant: *Domain of Interactions*). At the same time, self-identity in the autonomous individual (left side: *Autonomous Individual* insert) requires larger-world interactions that produce contextualized meanings about gender/sex (right side insert: *Individual in Interaction with Others and Objects*). As indicated by the large top arrow that links the Individual with the World, individuals cannot separate or articulate an understanding of self, outside of their location in the world’s meanings.

Such contextualized meanings may be thought of as gender schema (Liben and Signorella, 1980; Martin and Halverson, 1981) that provide (as discussed in section “Big Theory From Other Fields”) a generative model of gender/sex. The domain of interactions (upper right quadrant) starts with the absorption of bodily information as a subunit of the dyad (Beebe and Lachmann, 1994). Over time, the interactive domain expands to include interactions such as choice of clothing, toys, and peer interactions. These social interactions gain significance (Figure 2: lower right quadrant) as others interpret them as gender/sex. As infants observe positive, negative, or neutral valences attached to their own and others’ gender/sex representations, they feed (or link) this understood significance into a self-sustaining (autopoietic) gender/sex identity system via the intentional behaviors involved with self-socialization (Varela, 1997), understood as a child’s active efforts to match their own behaviors to a perceived standard (Zosuls et al., 2009, 2014; Tobin et al., 2010).

The emergence of intentional behavior moves the child from the domain of “Significance in the World” to the domain of “Operational Closure in the Individual” (Figure 2: lower left quadrant). In terms of gender/sex, we define operational closure as the multi-month process during which children acquire linguistic labels, the ability first to label gender/sex of self and others passively, then actively, over time acquiring the concepts of gender/sex constancy, and gender/sex stability (Fagot et al., 1985, 1986, 1992; Bem, 1989; Fagot and Leinbach, 1989, 1993; Fagot and Hagan, 1991). According to Varela, operational closure gives rise to a global property (what we call identity) without requiring “a central controller” such as an identity gene or a special group of identity brain cells.

Operational closure closes the autopoietic loop and stabilizes individual identity (upper left quadrant). Figure 2 represents identity as both a property of the individual body/mind *and* a collective property involving interactions with others and with objects in the world. While identity may appear to be a “thing,” it is actually a stable set of processes. Its development and continued maintenance and shaping depends on underlying activities that are both autonomous and intersubjective. Consider, for example, the common view of “properly” gender-identified boys and girls. The stereotypical boy runs around shooting a pretend gun and engages socially by chasing and running. The stereotypical girl plays quietly and engages in face-to-face social activities. Children in these idealized categories also prefer different clothes (Maccoby, 1998). Through these physical and interactive presentations, that vary continuously rather than in the stereotypical binary fashion so often presented, they come to understand themselves as a boy or a girl. They reinforce a blooming sense of identity by the very activities and codes of dress and conduct that led them to self-label in the first place. [On gender as process in adults see West and Zimmerman (1987), West and Fenstermaker (1995)].

During Phases 1 and 2 an infant’s gender/sex-related neuro-muscular and sensorimotor repertoires narrow, focus, and link to gender/sex in the world. In Phase 1, daily, moment-to-moment dyadic interactions are the crucial intermediaries connecting developing neural networks to “gender-in-the-world.” As Phase 2 blends into Phase 3, the neural networks that mediate “gender-in-the-world” reverberate as gender/sex identity in the toddler’s individual mind/body. This model is compatible with the idea that gender/sex expression and identity are interlaced continua. Through a variety of institutions, we usually force gender/sex identity and expression into a social and structural binary. For example, we only offer two possibilities on a birth certificate, two types of bathrooms, and until recently, children had only two identity options–boy or girl. To fit a continuum into a binary structure, researchers produced the concepts “gender non-conforming” or “gender variant.” In contrast, I hypothesize that the range of individual infant, parent, and infant-parent dyad differences in motor (and probably other) behaviors shapes a range of gender/sex embodiment. Such shaping ultimately feeds into the stream of information out of which identity itself coalesces. If this is so, the behaviors currently labeled and measured as “gender non-conforming, gender variant, gender atypical or gender incongruent”–all phrases widely used in the psychological research literature to describe non-binary presenting children- simply fall among a number of possible gender/sex identities (Zucker and Wood, 2011; Drescher et al., 2016).

## The Challenges: Synthesizing Theories and Investigatory Approaches to Explicating Gender/Sex Identity Development

The offered dynamic systems theory of gender/sex development draws on findings from a range of disciplines These disciplines focus on levels of organization ranging from the cellular to the socio-cultural. This brings us to a remaining set of questions–how can we elaborate and specify this dynamic developmental account and accumulate empirical data to elaborate the details while repeatedly and bidirectionally crossing boundaries of scale? How can we figure out which bits are supported by new data and improved theory and which will turn out to be wrong, and how can we project the relationships between levels of organization of changes that happen within any one level?

### Big Theory From Other Fields

Theoretical biologists and those who are trying to make sense of newly available large data sets are currently thinking and writing about how to traverse levels of organization. In this section I describe some of this work and point out ways in which it might translate to studying the dynamics of gender/sex development.

Rather than searching for causal links between evolution and development–events which happen on very different timescales-, Fields and Levin turned to “the language of communication, inference and information processing” (abstract) (Fields and Levin, 2020). Fields and Levin wrote that “The representation of organisms as active agents embedded in an interaction with active environments requires a reconceptualization of inheritance as the transfer across time not of a genome or other isolated memory-bearing structure but of …a living cell in continuous interaction with the environment” (pp. 4–5). In the following sentences I apply the structure of their argument to gender/sex development: In Phase 1 an infant is an active agent embedded in continuous interactions with active environments. These include the physical environment, the dyadic interactions with a caregiver and others, and the cultural environment within which the caregiver and others make behavioral choices. The infant encodes memories of repeated sensory events in its body- in the neuro-motor and autonomic nervous systems. These memories are comprised of continuously firing individual cells and collective neural activity rather than genes or genetic causes. During Phase 2, cellular- and organ-level memories begin to translate into cognitively accessible memories and emerge during Phase 3 as behaviors and subjectivity. During Phase 3 subjectivity stabilizes but, quoting Fields and Levin, it is not an “isolated memory bearing structure.” Nor is identity located somewhere specific. Rather it is the collective property of all the events depicted in Figure 1. One implication of this approach for neuroimaging studies might be that researchers look for neural network activity under circumstances designed to challenge or modulate felt identity.

I conceptualize identity as a process rather than a thing. A process theory posits that identity self-organizes rather than being built according to a genetic blueprint. Nor is identity a fixed trait. Once stabilized it remains a dynamic entity, held more or less constant by a continuous back and forth between supporting experience and embodied responses. The fact that external experience and social context sustains and shapes identity means that it is fundamentally intersubjective rather than individual and autonomous.

In treating gender/sex as a self-organizing system, I draw from a large biological literature on self-organization (Barabási and Albert, 1999; Camazine et al., 2001). The recent work of Yufik and Friston (2016) which explores cognitive understanding as an emergent property is of particular interest. They distinguish simple recognition (in my theory, for example, an infant’s ability to recognize gender/sex in voices or faces) from abilities that demand what they call a “generative model.” Yufik and Friston explain that a generative model of a circle (for example) entails not only the ability to visually recognize a circle, but also to imagine or perform manual circular manipulations, to walk in a circle), etc. “These abilities,” they write, “require a generative model… distinct from simply recognizing objects ….” In short, understanding is quintessentially enactive and “embodied,” requiring one to actively engage with the causes of sensations.“ (pp. 8–9) (I think that by age 3 most children have developed generative models of gender/sex). Earlier in this article, I discussed the idea of statistical chunks linking multimodal experiences as they relate first to the recognition and then to the understanding of gender/sex. Here, I raise the question of whether such chunks might be what Yufik and Friston call neuronal packets that form in associative networks and that maintain an internal integrity. To describe these they invoke the statistical concept of a Markov Blanket that links different chunks or nodes. Markov blankets stabilize the nodes they cover, provide them with a certain amount of statistical independence within a network, yet keep them connected to one another (Friston, 2011).

This may seem too abstract or even inappropriate for a discussion of gender/sex. Indeed, Friston’s work contains complex mathematical treatments of the statistical dynamics of semi-independent nodes and Markov blankets, a statistical concept that can link levels of organization. These mathematical treatments are beyond the reach of most students of gender/sex (myself included). But Yufik and Friston are working on a theory of embodied understanding, which is how I am trying to describe gender/sex. I believe that in response to a complex variety of sensory experiences, gender/sex concretizes in the body, specifically within the sensorimotor and autonomic nervous systems, and in behavior. Statistical frequencies and variations of specific experiences produce expectations based on the probability of a particular set of events. And events at one level of organization (say groups of nerve cells firing together) connect to others at different levels of organization (say a toddler demanding to put on pants or a dress). An academic discipline can be thought of as devoted to studying sets of associative networks within a particular level. To do interdisciplinary studies of the sort demanded by a multi-level theory of gender/sex requires a concept such as a Markov blanket that does the dual labor of both separating and linking different levels of analysis.

Finally, in thinking about how to move from one biological or developmental level of organization to the next, Delafield-Butt and Trevarthen take a non-mathematical approach by examining the early sensorimotor bases of the development of intersubjective and independent narrative (Delafield-Butt and Gangopadhyay, 2013; Delafield-Butt and Trevarthen, 2015). Although their proposed developmental trajectory from intentional sensorimotor movements *in utero* to the complex symbolic play of a toddler is linear, one could apply such a narrative analysis to non-linear developmental patterns. One might, for example, think of gender/sex as transforming from a precognitive narrative based on shared tasks that in infancy concern simple interactions (verbal narrative provided by the caregiver, movement and motor intentionality provided by the infant) to complex play involving movement, and conscious symbolism in toddlerhood. Such conceptualization, using the timeline presented in Figure 1, might provide a basis for future investigations.

### Some Interesting Methodology

Practically speaking, how can researchers capture, measure the frequencies and durations of individual and joint behaviors, and assess the importance for gender/sex development, of mundane events that happen repeatedly during the first year of life? And once having obtained such data, would it be possible to link it to the emergence of gender/sex subjectivity? To begin with (and quite obviously), I am proposing longitudinal studies that, ideally, must last for at least 3 years (from birth to the acquisition of a preliminary gender/sex identity). Even better would be to weave into the study design the ability to check in on study subjects during mid childhood, puberty and late adolescence. Studies of this length are rare, but possible (Merrick, 2013).

In her short film of bathing infants in three cultures, Margaret Mead pioneered the use of narrated episodic, *in situ* observational recording for the study of infant development (Mead, 1940). During the 1960’s researchers attempted to quantify naturalistic in-home studies using a multiple input keyboard attached to a mechanical event recorder (Moss, 1967). The quantitative analysis of film-based videos and finally of digital recordings followed in subsequent decades. But quantifying visual records is extraordinarily time consuming, requiring researchers to limit the number of study subjects and/or at great expense, hire a large number of human data analysts. In the past couple of decades, however, automated data recording has become available.

The LENA system, for example, provides automatic language recording, monitoring and analysis that can be used to examine vocal interactions between care-givers and infants. In one study of 16 h long interactions, Johnson et al. reported that infants from birth through 7 months experienced more female than male adult speech. Adult women responded more often to infant vocalizations and infants responded more to adult female, compared to adult male speech (Johnson et al., 2014). In a tour de force of what they refer to as “dense data collection” Roy and colleagues documented language development by recording a complete record of sounds and words made by a single child during his first 3 years of life (Roy et al., 2015). They concluded that mere frequency of word repetition was less important for language learning than the location in which a word was spoken, as well as the time of day and the ways in which a particular word was embedded in the context of everyday speech. As fascinating as the Roy et al. study is [see also (Roy et al., 2006)], their methods present difficulties for widespread adoption. One compromise between a totally and intrusively wired home environment and artificially structured laboratory experiments is to study free play in a home-like environment that has multiple camera angles and sensors distributed in the room. Yu (2020) created such an environment in order to study coordinated parent-infant social interactions, and a variety of multimodal parental effects on infant visual attention. With some thought, a multiply wired home-like environment that can record interactions from several points of view, could be adapted to the study of gender/sex, provided it was coupled with an assessment of the actual home environment (home visits to assess physical and toy environment, questionnaires designed to assess gender/sex beliefs of caregivers, etc.).

Automated data collection and analysis has become part of the next wave of infant study. de Barbaro proposes best practices for the use of wearable sensors to record motion, autonomic function and vocalization within what she calls the ecology of daily activity (de Barbaro et al., 2013b; de Barbaro, 2019). She also advocates for the study of unstructured home-based activities. de Barbaro notes that many aspects of natural activity are not present in carefully structured laboratory activities which are, in the first place, designed to limit the number of study variables. Wearable sensors permit the collection of large volumes of data, recording activity over varying timescales, thus allowing the potential analysis of phenomena that develop over hours, days, weeks, and months. Although de Barbaro and colleagues have not applied their approach to the study of gender/sex, there is no reason to think that gender/sex differs fundamentally from other developmental phenomena; I argue that this extensive new methodology be applied to the study of gender/sex development rather than continuing to study children in over-simplified settings with a stripped down number of study variables.

de Barbaro discusses the several methodological challenges to embracing these new technologies and to reopening the study of development to long-term open-field conditions. These, of course, require attention, but in this essay I want to emphasize the direction the field ought to take rather than offer reasons for why a new path cannot be developed. One last note about technology. Just as de Barbaro champions wearable sensors that can detect movement, emotion and interpersonal interactions, the work of Kuhl and her colleagues demonstrates that wearable sensors can reach “down” into the brain for the analysis of brain activity correlates of specific behaviors (Kuhl et al., 2001; Kuhl, 2004, 2010; Kuhl and Rivera-Gaxiola, 2008). Thus, interdisciplinary study designs that reach “down” into the body but also out into the surrounding world are within reach.

**FIGURE 3:**
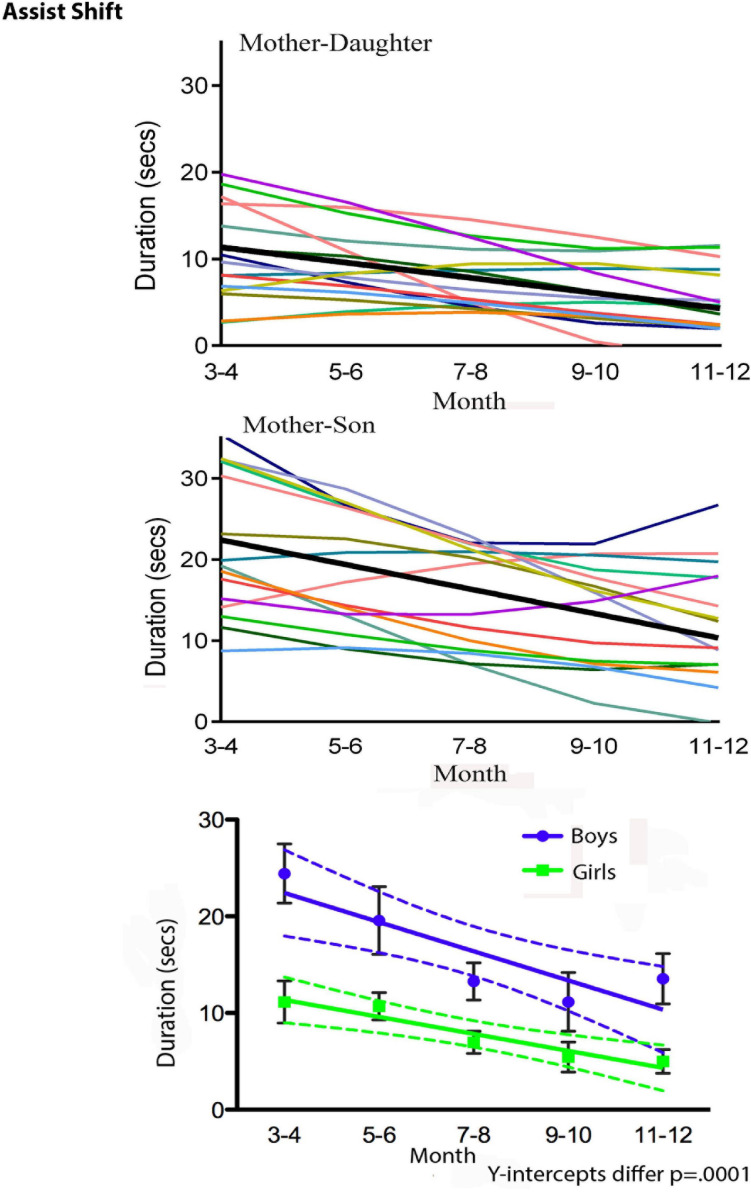
Duration of Assist Shift in 2-month groups from 3 to 12 months. Top panel shows individual dyads with girls (thin colored lines) and a regression line for dyads with girls (thicker black line). Middle panel shows individual dyads with boys (thin colored lines) and a regression line for dyads with boys (thicker black line). The bottom panel shows group regression lines and standard deviations (square symbol, green line = mother-daughter dyads; round symbol-blue line = mother-son dyads).

**FIGURE 4:**
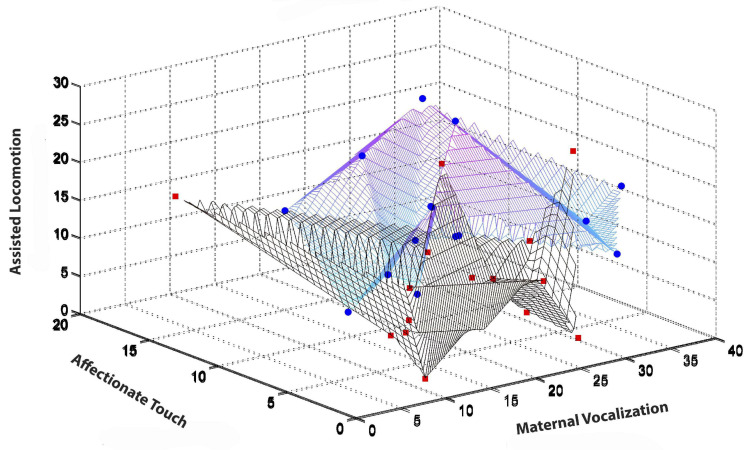
Illustrates the 3D state space occupied at 3–4 months of infant age for the maternal behaviors of assisted locomotion (*Y* axis), affectionate touch (*X* axis), and maternal vocalization (*Z*-axis). Two types of graphs–a mesh and a scatterplot are overlaid. The blue circles (mother-son) and red squares (mother-daughter) represent individual mother-infant dyads. The black-lined mesh represents the state-space occupied by mother-daughter dyads, while the blue-lined mesh represents the state-space occupied by mother-son dyads. Color variation (from blue to purple) results from mesh density.

The turn to dense, multimodal, and longitudinal data collection also requires new types of statistical analysis. de Barbaro reviews a number of these, including visualization techniques such as state-space grids (Hollenstein, 2007, 2013), and statistical modeling of use for analyzing dense, repeated measures data. Figure 3 illustrates, for the purpose of example, some results from Fausto-Sterling et al. who used a form of longitudinal analysis developed by Singer (Singer and Willett, 2003; Fausto-Sterling et al., 2020). The graph illustrates gender/sex differences over time in maternal shifting of the infant from one location to another (Fausto-Sterling et al., 2020). This graphical presentation has the advantage of showing group differences (illustrated in the bottom panel of Figure 3) while also allowing the visualization of within-group individual differences. We can also visualize the statistical distribution of multiple behaviors using three dimensional visualizations. Figure 4 is a three dimensional state-space graph of three maternal behaviors, affectionate touch, assisted locomotion and maternal vocalization in mother-son and mother-daughter dyads when the infants were 3 to 4 months of age. This representation allows the viewer to look at individual data points while also using the mesh blanket to conceptualize the idea of 3-dimensional state spaces for combinations of maternal behavior.

In a different approach, Eason and colleagues explored relationships in mother/infant interactions using vector autoregression analysis. This method promises an approach for testing gender/sex salience in multimodal, bidirectional effects in a dense, multimodal data set (Eason et al., 2020). Finally, both biologists and cognitive scientists are exploring the use of Bayesian statistics in the longitudinal study of development. Kuchling et al. developed Bayesian models to explore how small groups of cells assess their individual and collective states and predict their own forward-looking genetic and physiological activities based on their reading of the environment created by other small groups of surrounding cells. The developmental goal is to cooperate in achieving complex pattern formation and morphogenesis. In this model, cells use Bayesian inference, which is a statistical process in which cells update their prior physiological state based on contemporary sensing of their current environment (Kuchling et al., 2020). Directly germane to cognitive development, Gopnik champions the use of Bayesian methods to explore how children derive and build cognitive theories, and it should be worthwhile to apply such methods to the development of children’s theories about gender/sex (Gopnik, 2010; Gopnik and Bonawitz, 2015).

**FIGURE 5:**
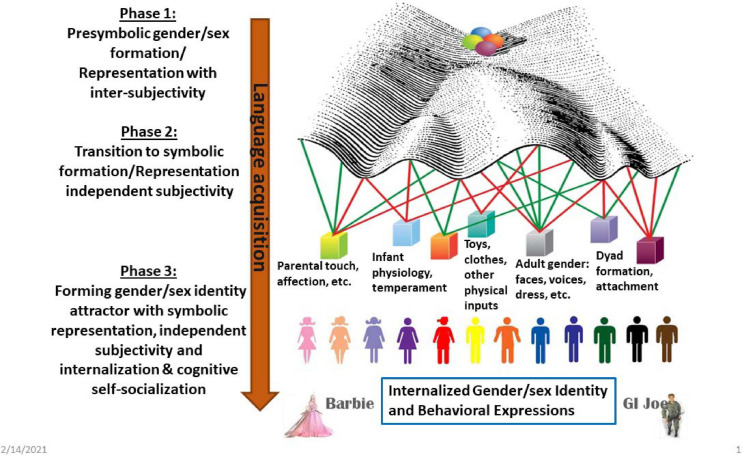
The left side of the diagram reiterates the phased development of gender/sex from intersubjective to subjective. The right side visualizes the process as a modification of a Waddington-style genes and landscape drawing. The drawing represents Waddington’s guy-wires as swinging weights representing landscape-shaping inputs ranging from physiology to culture, and producing a continuum of gender/sex. One might imagine that Phase 1 correlates with the initial start of the balls rolling downhill and probably includes the initial bifurcation in the landscape. Phase 2 might be seen as starting with the secondary bifurcations (drawing by the author).

Visualization can help us understand developmental complexity, including transitions along widely varying time scales and between levels of organization that range from cells to behaviors to subjective psychology. By doing a deep dive into C.H. Waddington’s famous drawing of epigenetic landscapes, feminist science studies scholar Susan Squier explored drawing as metaphor. Waddington’s illustrations, she argued, entail “productive engagements with the unknown” (p. 17) (Squier, 2017). Baedke reviewed some of the ways in which Waddington’s drawings contributed to the development of new knowledge in fields of study ranging from applied mathematics to developmental psychology (Baedke, 2013), while Flower explores the use of visualization to understand the dense data produced by studies that produce individual molecular profiles for thousands of single cells (Flower, 2020). As an example, Figure 5 uses a modification of one of Waddington’s drawings to visualize events described in Figure 1. In the original, balls (i.e., organisms) rolled down the landscape reaching final phenotypes. Waddington imagined genes as fixed guy-wires that shaped the developmental landscape by pulling from underneath. The redrawing imagines the pulls as swinging weights representing landscape-shaping inputs ranging from physiology to culture. This visualization emphasizes dynamic movement, although it is still not quite right because the possible effects of the developing organism on the weights themselves as well as development over the life cycle are not properly illustrated.

### Interdisciplinary Collaboration

How can a researcher trained primarily in a particular discipline possibly accomplish such long term and multidisciplinary tasks? The answer: through collaborative consortia such as the Many Baby Project (MBP) or the Baby Connectome Project (Bergmann et al., 2020; Elison, 2020). The Many Baby Project provides one possible starting model for a collaborative, interdisciplinary research consortium on gender/sex (Many Babies, 2021). Begun in 2016 with the express purpose of addressing the replication crisis in psychology, participants identified as a central aim to better understand why different labs that use similar methods get different experimental results. To further this aim MBP collaborators agreed to replicate a small number of findings on infant development that, based on metanalysis, seemed “true” even though individual reports did not always replicate the finding (Frank et al., 2017). They hoped also to increase the non-WEIRDness of their study sample, lessen the burden of large-scale data collection for any one lab, standardize study methods to make data more directly comparable from one site to the next, and use an open science framework to make raw data available for secondary analysis. Their operating principles included collective governance, inclusivity and diversity, and ethical research. The MBP participants provide a rich record of their process and online tools that could be adapted for the study of gender/sex in infancy (Bergmann et al., 2019).

However exciting, the MBP project does not venture into an interdisciplinary framework that traverses levels of biopsychological organization ranging from cellular function and physiology, to brain organization and function, to individual and dyadic behavior patterns and subjectivity. To accomplish such multilevel analyses participants must learn to have interdisciplinary conversations. The goal is to figure out how to draw conclusions that translate across levels of organismic organization (and disciplinary boundaries). Like the theory itself, the collaboration must focus on emergent rather than additive developmental models.

A group consisting of individuals from disciplinary backgrounds ranging from feminist philosophy of science, to experimental neuroimaging modeled such an effort by having an interdisciplinary conversation about strongly believed-in findings of gender/sex differences in spatial abilities in adults (Bentley et al., 2019a, b). Initially they had hoped to agree on an experimental design that studied gender/sex and spatial abilities across levels of organization from the hormonal to the social. They began, as any inter-disciplinary conversation must, by making explicit their own causal models and clarifying their varied uses of the sex and gender terms. As they worked toward a common language for underlying theories of experimentation and of gender/sex, they diagrammed variables of interest and illustrated what they called their entanglements [see also (Fausto-Sterling, 2000), pp. 141–143]. As their discussion proceeded, Bentley et al. (2019b) realized that a single protocol could not accommodate all of the issues they had raised, and so they chose to “showcase …a negotiation process–an aspect of collaborative research that usually remains a non-public affair…” p. 2/20 (Bentley et al., 2019b). I cite this effort not because it succeeded in its initial goal, but because it was a first attempt. We need more conversations of this sort aimed at devising empirical and theoretical investigations into gender/sex identity formation.

## Cheerleading and Summary

We can take advantage of new developments in the brain sciences and in the study of infant development to investigate gender/sex as it emerges in toddlers. The use of wearables that record neural activity and physiological change, and of automated recording of individual and dyadic behaviors, and the development of theory aimed at understanding moments of transition and the establishment of stability provide the potential to achieve new understandings of the early development of gender/sex. As complex as such a project might be, it seems worth it for several reasons. First, it would be nice to do better science, to move in a positive way toward understanding gender/sex variability. Second, getting the science right (or at least better) can help with the development of sensible health and social policy having to do with the development of job opportunities and better health care for the full range of gender/sexed humans (Şahin and Soylu Yalcinkaya, 2020).

## Ethics Statement

The studies involving human participants were reviewed and approved by the Brown University Institutional Review Board. A copy of the original consent form may be obtained from the author.

## Author Contributions

The author confirms being the sole contributor of this work and has approved it for publication.

## Conflict of Interest

The author declares that the research was conducted in the absence of any commercial or financial relationships that could be construed as a potential conflict of interest.
